# Supplementary material for: Transcriptomic Analysis Reveals Host miRNAs Correlated with Immune Gene Dysregulation during Fatal Disease Progression in the Ebola Virus Cynomolgus Macaque Disease Model
Source: Microorganisms. 2021 Mar 23;9(3):665. doi: 10.3390/microorganisms9030665 (PMC8005181; doi:10.3390/microorganisms9030665)
Supplement: Supplementary file 1 [file microorganisms-09-00665-s001.zip › microorganisms-1142619-SI.pdf]

Transcriptomic analysis reveals host miRNAs correlated with immune gene dysregulation during fatal disease progression in the Ebola virus cynomolgus macaque disease model

Christopher P. Stefan [1∞], Catherine E. Arnold [1∞], Charles J. Shoemaker [1∞], Elizabeth E. Zumbrun [2], Louis A. Altamura [1], Christina E. Burrows [1], Cheryl L. Taylor-Howell [1,] Amanda S. Graham [1], Korey L. Delp [1], Candace D. Blancett [1], Keersten M. Ricks [1], Scott P. Olschner [1], Joshua D. Shamblin [2], Suzanne E. Wollen [2], Justine M. Zelko [2], Holly A. Bloomfield [2], Thomas R. Sprague [2], Heather L. Esham [2], and Timothy D. Minogue [1*]

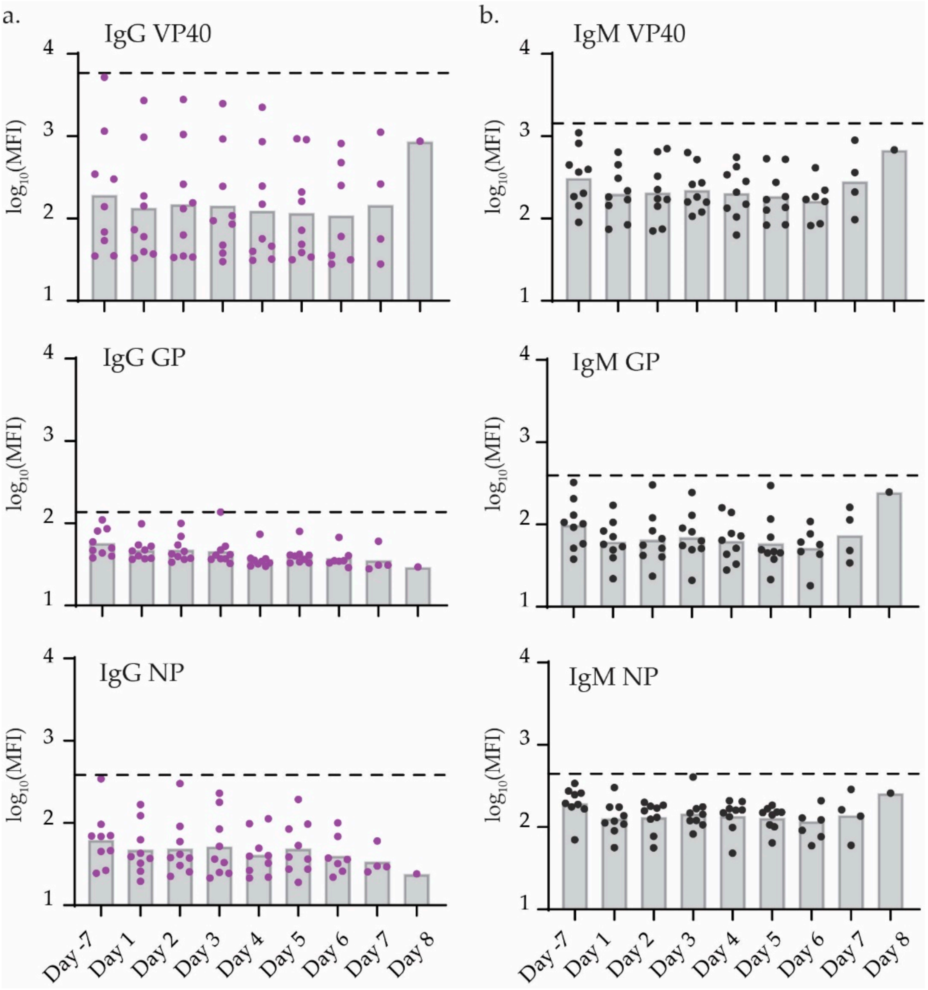

Figure S1. IgG and IgM response in Ebola infected NHPs. (a) IgG and (b) IgM MFI serum concentrations measured using recombinant EBOV GP, NP, and VP40 challenged for each NHP across all times points. For all graphs dots represent one NHP sample while bars indicate the mean of all samples. The average of day -7 samples plus three standard deviations were used as a cutoff and represented by a dashed line.
